# Prebiotic Oligosaccharides in Skin Health: Benefits, Mechanisms, and Cosmetic Applications

**DOI:** 10.3390/antiox14060754

**Published:** 2025-06-18

**Authors:** Meijun Zeng, Yang Li, Jie Cheng, Jingyu Wang, Qiyu Liu

**Affiliations:** 1 Meat Processing Key Laboratory of Sichuan Province, College of Food and Biological Engineering, Chengdu University, Chengdu 610106, China; zengmeijun@cdu.edu.cn (M.Z.); m19923603356@163.com (Y.L.); chengjie@cdu.edu.cn (J.C.); 2 National and Local Joint Engineering Laboratory of Energy Plant Biofuel Preparation and Utilization, College of Chemistry, Sichuan University, Chengdu 610064, China; 3 Guangdong Provincial Key Laboratory of Plant Resources Biorefinery, School of Chemical Engineering and Light Industry, Guangdong University of Technology, Guangzhou 510006, China

**Keywords:** non-digestible oligosaccharides, pathogen inhibition, skin diseases, skin barrier function, commercial cosmetics

## Abstract

Prebiotic oligosaccharides have attracted significant interest in dermatology and skin health due to their ability to modulate the skin microbiome and microbiota–host interactions. This review offers a novel dual perspective, systematically examining the benefits of both oral intake and topical application of prebiotic oligosaccharides, including well-established prebiotics (e.g., human milk oligosaccharides, galacto- and fructo-oligosaccharides) and emerging prebiotic candidates (e.g., gluco-oligosaccharides, chitosan-oligosaccharides, agaro-oligosaccharides). First, cutting-edge synthetic processes for producing diverse oligosaccharides and their structural chemistry are introduced. Then, we discuss in vitro studies demonstrating their efficacy in promoting skin commensals, inhibiting pathogens, and conferring protective effects, such as antioxidant, anti-inflammatory, anti-melanogenic, and wound-healing properties. Furthermore, we emphasize in vivo animal studies and clinical trials revealing that prebiotic oligosaccharides, administered orally or topically, alleviate atopic dermatitis, enhance skin hydration, attenuate acne, and protect against photo-aging by modulating skin–gut microbiota and immune responses. Mechanistically, we integrate genetic and molecular insights to elucidate how oligosaccharides mediate these benefits, including gut–skin axis crosstalk, immune regulation, and microbial metabolite signaling. Finally, we highlight current commercial applications of oligosaccharides in cosmetic formulations while addressing scientific and practical challenges, such as structure–function relationships, clinical scalability, and regulatory considerations. This review bridges mechanistic understanding with practical applications, offering a comprehensive resource for advancing prebiotic oligosaccharides-based skincare therapies.

## 1. Introduction

The skin microbiota, the human body’s second-largest microbial community after the gut, plays a crucial role in skin disease and well-being [1,2]. Skin microbiota can be commensal, mutualistic (beneficial), or pathogenic. A balanced composition of commensal (e.g., *Staphylococcus epidermidis*, *Cutibacterium acnes*) and pathogenic (e.g., *Staphylococcus aureus*) microorganisms maintains skin homeostasis [3], while dysbiosis contributes to disorders like atopic dermatitis, acne, psoriasis, etc. [4,5,6,7]. In addition, the skin microbiota also actively supports skin barrier function, including skin hydration, anti-aging, anti-melanogenesis, etc. [1,8].

Prebiotics have recently gained significant attention in dermatology and skin health due to their ability to modulate host microbiota beyond the gastrointestinal tract [9,10]. Defined as substrates selectively utilized by host microorganisms to confer health benefits, prebiotics now encompass compounds targeting diverse microbial communities, including those residing on the skin [11,12,13]. While dietary fibers, like fructans, carrageenan, and fucoidans, have been extensively reviewed for skin health benefits [14,15,16], prebiotic oligosaccharides, which usually consist of 2–20 monosaccharide units, represent particularly promising skin therapies. Well-established prebiotic oligosaccharides include human milk oligosaccharides (HMOs), galacto-oligosaccharides (GOSs), and fructo-oligosaccharides (FOSs), which show efficacy in mitigating atopic dermatitis [17,18,19]. Emerging oligosaccharides, such as gluco-oligosaccharides (GlcOSs), xylo-oligosaccharides (XOSs), chitosan oligosaccharides (COSs), agaro-oligosaccharides (AOSs), etc., exhibit diverse biological activities, such as antioxidant, anti-inflammatory, and anti-aging effects, as evidenced by in vitro and in vivo studies [20,21,22].

Current research on prebiotic oligosaccharides has primarily investigated their oral administration and systemic effects on skin health. But accumulating evidence now demonstrates the efficacy of topical application in delivering targeted dermatological benefits [18,23]. Clinical studies have documented that GOSs incorporated in cosmetic formulations significantly enhance skin hydration, particularly improving water-holding capacity and reducing transepidermal water loss in healthy female subjects [24,25]. Furthermore, emerging clinical evidence supports the anti-aging and photoprotective properties of various functional oligosaccharides when applied topically, including GlcOSs [26], XOSs [21], and COSs [27]. These findings highlight the dual mechanisms of oligosaccharides through oral intake or topical application, underscoring their considerable potential as active ingredients in advanced cosmetic formulations, which aligns with the growing consumer demand for science-backed, bioactive ingredients for next-generation skincare products.

Despite substantial evidence supporting the skin health benefits of functional oligosaccharides, significant knowledge gaps remain regarding their mechanisms of action. For example, oral administration of HMOs has been shown to alleviate pediatric atopic dermatitis (AD) through gut microbiota modulation and immune system interactions [17], but the underlying biological pathways require further elucidation. Similarly, in vitro and in vivo studies observed benefits of GOSs, FOSs, and GlcOSs in acne management, skin hydration, anti-aging, etc. [28,29,30]. However, methodological inconsistencies across studies and insufficient investigation of the structure–biological function relationships of oligosaccharides significantly limit the basic understanding of their mechanisms of action. Particularly, the underlying mechanisms of topically applied prebiotic oligosaccharides remain poorly understood because of the scarcity of dedicated studies.

**Figure 1:**
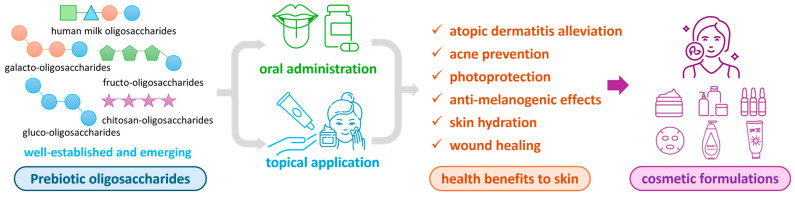
Illustration of prebiotic oligosaccharides through oral administration or topical application to confer health benefits to the skin and application in cosmetic formulations.

Therefore, this review comprehensively summarizes recent advances in the applications of well-established and tentative prebiotic oligosaccharides—both orally and topically—in modulating skin microbiota and their subsequent effects on skin health and cosmetic benefits (illustrated in Figure 1). These benefits include, but are not limited to, the alleviation of atopic dermatitis, acne prevention, enhanced skin hydration, anti-melanogenesis, anti-aging properties, and photoprotection. First, well-established and tentative prebiotic oligosaccharides are introduced, covering their chemical structures and novel production methods. Next, we systematically review in vitro and in vivo studies elucidating the functional roles of oligosaccharides in skin health, followed by an in-depth discussion of their underlying mechanisms of action. Additionally, this review examines the practical utilization of prebiotic oligosaccharides in commercial cosmetic formulations, emphasizing key bioactive ingredients and their integration into skincare products. Finally, we address current challenges and propose future research directions to advance this promising field.

## 2. Prebiotic Oligosaccharides: Chemical Structures and Novel Production Methods

The International Association of Probiotics and Prebiotics (ISAPP) defines prebiotics as substrates selectively utilized by host microorganisms to confer health benefits, including those targeting extraintestinal niches like the skin [12]. While formally recognized prebiotics include non-digestible oligosaccharides, such as human milk oligosaccharides (HMOs), fructo-oligosaccharides (FOSs), and galacto-oligosaccharides (GOSs), emerging candidates, like gluco-oligosaccharides (GlcOSs), xylo-oligosaccharides (XOSs), and chitosan oligosaccharides (COSs), are gaining plenty of attention. This section summarizes the skin-beneficial oligosaccharides, detailing their structural characteristics (monomeric composition, degree of polymerization, glycosidic linkages) and production methods (Table 1), as these parameters critically determine their skin health efficacy.

### 2.1. Human Milk Oligosaccharides (HMOs)

**Figure 2:**
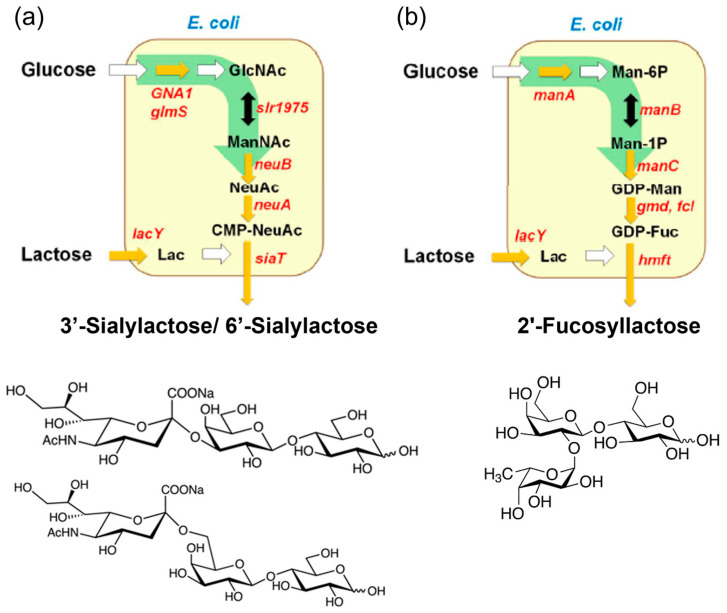
Industrial enzymatic production of human milk oligosaccharides. Biosynthetic pathway of (**a**) 3′-sialylactose/6′-sialylactose and (**b**) 2′-fucosyllactose from glucose and lactose (Lac). Sugar nucleotides, such as cytidine 5′-monophospho-N-acetylneuraminic acid (CMP-NeuAc) and guanosine diphosphate fucose (GDP-Fuc), are biosynthesized internally from glucose, and glycosyltransferases transfer the sugar moiety of the nucleotide sugar to lactose. (Reproduced with permission from [31]). Notes: GlcNAc: N-acetylglucosamine; ManNAc: N-acetylmannosamine; NeuNAc: N-acetylneuraminic acid; Man-6P: mannose 6-phosphate; Man-1P: mannose 1-phosphate; GDP-Man: guanosine pyrophosphate mannose.

The first recognized group of prebiotics includes HMOs, with approximately 200 different structures characterized in human breast milk [12,32]. Structurally, HMOs consist of fucose (Fuc) and/or N-Acetylneuraminic acid (Neu5Ac) attached to galactose, N-Acetylglucosamine (GlcNAc), or glucose residues, classifying them into fucosylated, sialylated, and neutral core HMOs [51]. For research purposes, HMOs can be isolated directly from breast milk or synthesized through enzymatic, chemical, or chemoenzymatic methods. Enzymatic methods catalyzed by glycosidases, glycotransferases, or glycosynthases enable the production of diverse HMOs with varying DPs, monomer compositions, and glycosidic linkages [32]. Commercial-scale production of HMOs relies on microbial fermentation techniques, where metabolically engineered microorganisms (primarily *E. coli*) convert inexpensive carbon sources, such as lactose and glucose, into HMOs [31,32,33]. As illustrated in Figure 2, the strains are modified to express glycosyltransferases and synthesize sugar nucleotides (e.g., CMP-Neu5Ac, GDP-Fuc, UDP-GlcNAc) in vivo. The glycosyltransferases then transfer sugar moieties (e.g., sialy group, fucosyl groups) to lactose, yielding HMOs, such as 3′-sialylactose/6′-sialylactose and 2′-fucosyllactose (2′-FL) [13]. Notably, 2′-FL, the most prevalent HMO, has been produced commercially and widely incorporated into infant formula [52].

### 2.2. Galacto-Oligosaccharides (GOSs)

**Figure 3:**
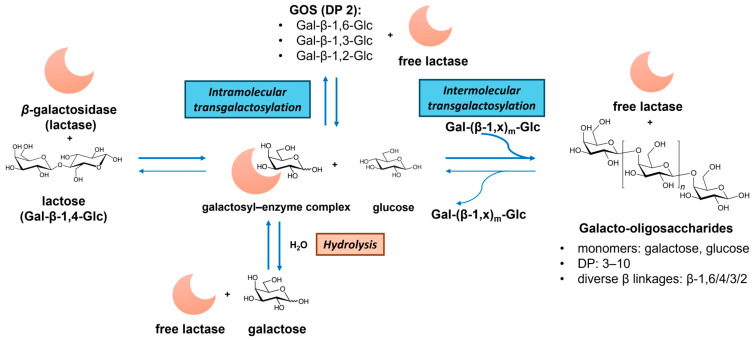
Illustration of the enzymatic production of galacto-oligosaccharides (GOSs) from lactose via hydrolysis and transgalactosylation reactions catalyzed by β-galactosidase (lactase). Note: DP—degree of polymerization.

GOSs consist of galactosyl oligomers typically terminating with lactose. Commercial GOS products, such as Vivinal^®^ GOSs, Bimuno^®^ GOSs, and Oligomate 55^®^ GOSs, are produced via β-galactosidase (EC 3.2.1.23, from, e.g., *Bacillus circulans*, *Streptococcus thermophilus*, *Bifidobacterium bifidum*, and *Aspergillus oryzae*)-catalyzed transgalactosylation of lactose [34,36]. As illustrated in Figure 3, GOS synthesis involves lactose hydrolysis, galactosyl–enzyme intermediate formation, and intra- and intermolecular transgalactosylation. The product yield and structures depend on the kinetic difference between hydrolysis and transgalactosylation, which are governed by reaction conditions (e.g., initial lactose concentration, temperature, water activity) and enzyme properties (hydrolytic activity and transferase activity) [53]. The resulting GOS mixtures exhibit diverse DP (usually 2 to 10) and glycosidic bonds (β-(1→6), β-(1→4), β-(1→3), β-(1→2), and also (1↔1)-linked non-reducing disaccharides (β-D-Gal*p*-(1↔1)-α-D-Glc*p* and β-D-Gal*p*-(1↔1)-β-D-Glc*p*)) [54]. Alternative dairy sources, like milk and whey permeates, have been utilized to enhance the GOS production economy [53,55]. A recent advance involves the chemical synthesis of GOSs via lactose hydrolysis and dehydrative glycosylation in concentrated sulfuric acid, generating potential prebiotic GOSs with novel α and β linkages [35].

### 2.3. Fructo-Oligosaccharides (FOSs)

**Figure 4:**
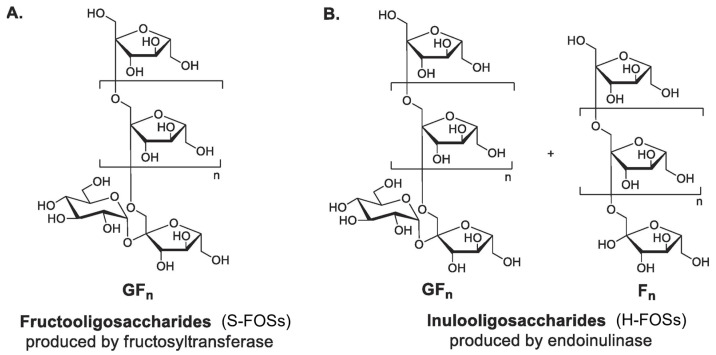
Chemical structures of two commercial fructo-oligosaccharides (FOSs) from different production methods. (**A**) S-FOSs produced from the enzymatic transglycosylation of sucrose; (**B**) H-FOSs produced from the controlled enzymatic hydrolysis of inulin (G and F denote glucose and fructose units, respectively. (Reproduced with permission from [56].)

FOSs consist of β-(2→1)-linked fructosyl chains terminating with sucrose. Commercial FOS production is well-established by major manufacturers (Actilight, Beneo, Meiji, NutraFlora) [37]. Two industrial processes yield structurally distinct FOSs (Figure 4). S-FOSs are produced via sucrose transfructosylation using fructosyltransferases (FTases), yielding exclusively GF_n_ type FOSs (1-kestose (GF_2_), nystose (GF_3_), and fructosylnystose (GF_4_)) [56]. Recent research focuses on the heterologous expression of FTases from yeast and bacterial expression systems [57]. On the other hand, H-FOSs are generated through controlled inulin hydrolysis by endoinulinase, producing mixed GF_n_ type and F_n_ type FOSs (such as inulobiose (F_2_), inulotriose (F_3_), and inulotetraose (F_4_)) [37,58]. H-FOSs exhibited broader DP distribution (from two to nine) and larger average DP (4.0) compared to S-FOSs (from two to four, average DP 3.6) [59]. In the lab, while acid hydrolysis (HCl/H_2_SO_4_) offers a simpler alternative for inulin hydrolysis: it generates undesired byproducts (fructose, 5-HMF) with low FOS selectivity [37].

### 2.4. Gluco-Oligosaccharides (GlcOSs)

GlcOSs comprise glucose oligomers with α/β-(1→6/4/3/2/1) glycosidic linkages. The predominant commercial GlcOSs are isomalto-oligosaccharides (IMOs), which are DP 2–6 GlcOSs with mainly α-(1→6) and α-(1→4) glycosidic bonds, marketed as ISOThrive nectar, Vitafiber, FiberYu, IMO-900, Wako IMOs, etc. [60]. As detailed in our recent review [40], industrial IMO production employs either (1) starch (corn, tapioca) processing via sequential liquefaction, hydrolysis, and transglycosylation using hydrolases and α-transglucosidase or (2) direct sucrose/maltose conversion via dextransucrase (EC 2.4.1.5)-catalyzed transglycosylation. A less common commercial GlcOS, gentio-oligosaccharides (GnOSs), consists of β-(1→6)-linked GlcOSs synthesized through β-glucosidase-mediated glucose reversion [61].

Recent advances in chemical synthesis have expanded the routes for producing prebiotic GlcOSs from glucose, maltose, and cellulose feedstocks. Dehydrative glycosylation of glucose in concentrated LiBr (61.7%), H_2_SO_4_ (≥72%), or H_3_PO_4_ (≥85%) yields short-chain GlcOSs (DP mostly 2–10) with diverse α/β-(1→6/4/3/2/1) linkages [62,63]. Recent advances in linkage-controllable GlcOS synthesis include zeolite-confined glucose glycosylation [64] and direct maltose glycosylation in unacidified LiBr [42]. Moreover, the production of β-(1→4)-linked cello-oligosaccharides (one type of GlcOS) from abundant cellulose resources is highly promising [65]. The key challenges involve the disruption of cellulose’s crystalline structure within weakly acidic solvents (nonacidified 61.7% LiBr (a.q.), essential for achieving selective cleavage of glycosidic bonds) [66,67] and controlled depolymerization to oligosaccharides (DP 4–11, 90.3% yield) while minimizing glucose formation [68]. To advance commercial viability, current research focuses on (1) developing efficient separation protocols for oligosaccharide recovery [69,70], (2) optimizing solvent systems for obtaining higher GlcOS concentration [71], and (3) employing combined hydrolysis and glycosylation to improve GlcOS solubility in water [72]. These methods have been explored on various lignocellulosic biomasses (e.g., birch, poplar, and corn stover) for GlcOS production, demonstrating potential for sustainable industrial production [45,73,74].

### 2.5. Other Prebiotic Oligosaccharides

Xylo-oligosaccharides (XOSs) are β-(1→4) linked xylose oligomers (DP typically 2–6), commercially produced from corncob xylan through alkaline extraction and controlled enzymatic hydrolysis by endo-1,4-xylanase. Recent efforts focus on utilizing abundant lignocellulosic biomass (e.g., wheat bran, cotton stalk, corn stover, sugarcane bagasse) as alternative feedstocks for XOS production [75,76]. The resulting XOSs maintain the β-(1→4) xylose backbone but may contain arabinose or ferulic acid substitutions, depending on the source material [77].

Chitosan oligosaccharides (COSs) are β-(1→4) linked glucosamine oligomers that are produced via chitin deacetylation and depolymerization processes. Acid hydrolysis (e.g., HCl, H_3_PO_4_), oxidative degradation, ultrasonic treatment, ultraviolet irradiation, and enzymatic hydrolysis (chitosanases, cellulases) are feasible for chitin depolymerization [47,48]. Deacetylation can be achieved using fungal-derived chitin deacetylase or chemically through a reaction in a concentrated alkaline solution (e.g., 50% NaOH) [78,79].

Agaro-oligosaccharides (AOSs) derive from agar (extracted from red algae) degradation via acid hydrolysis (HCl, H_3_PO_4_, citric acid, solid acids, trifluoroacetic acid), enzymatic hydrolysis (α/β-agarases), or microbial fermentation (e.g., *Bacteroides uniformis* NP1) [20]. AOSs contain two structural types: neoagaro-oligosaccharides (NAOSs) with α-1,3-linked-3,6-anhydro-L-galactose (L-AHG) termini and agaro-oligosaccharides (AOSs) with β-1,4-linked-D-galactose (D-Gal) termini.

## 3. In Vitro Studies of Prebiotic Oligosaccharides on the Skin Microbiome and Related Skin Health Effects

Table 2 highlights recent in vitro studies of prebiotic oligosaccharides (e.g., HMOs, GOSs, FOSs) and candidate oligosaccharides (e.g., GlcOSs, XOSs, COSs) on the promotion of skin health. The key findings include the positive effects of oligosaccharides on the promotion of skin commensal and pathogen inhibition, anti-melanogenic effects, antioxidant and anti-inflammation, and wound healing. In the following subsection, the specific type, chemistry, and dosage of oligosaccharides are introduced, and the action of mechanisms by which they confer skin health effects are discussed as well.

### 3.1. Promotion of Skin Commensals and Inhibition of Pathogens (HMOs, GOSs, FOSs, GlcOSs)

Mortaz et al. reported that galacto-oligosaccharides (GOSs) and 2′-fucosyllactose (2′-FL) directly suppressed the growth of specific pathogenic microbes, i.e., *Staphylococcus aureus* (SA) and *Pseudomonas aeruginosa* (PA), and affected the phagocytosis of neutrophils [80]. Their results showed that GOSs (5%, 10%) and 2.5% 2′-FL significantly decreased SA and PA bacterial growth/CFUs (*p* ≤ 0.05), and SA and PA growth lag times were suppressed by co-incubation with GOSs (>2.5%) or 2′-FL (1.5%). In vitro assays using human blood polymorphonuclear cells indicated that GOSs/2′-FL were able to increase the ability of bacteria phagocytosis by neutrophils, providing potential wound healing.

Early in vitro studies indicated substantial metabolism of gluco-oligosaccharides (GlcOSs) by skin commensals, such as *Rothia kristinae*, *Kytococcus sedentarius*, *Staphylococcus capitis*, *Corynebacterium xerosis*, and *Lactiplantibacillus pentosus* [97]. In contrast, pathogenic or opportunistic strains, such as *Staphylococcus aureus*, *Gardnerella vaginalis*, and *Cutibacterium acnes*, did not or minimally metabolized the GlcOSs. Meanwhile, the acidic medium (potentially lactic acid) after bacterial fermentation also showed an inhibitory effect on skin pathogens. Another study reported that GlcOSs alone inhibited glycocalyx production by *S. aureus* cells, indicating their ability to suppress the skin pathogen [83].

Liu-Walsh et al. reported that a colloidal-oat-containing prebiotic β-glucan (5%) significantly promoted the growth rate and metabolism of the skin commensal *S. epidermidis* ATCC 12228, while it only increased the growth rate of *S. aureus* ATCC 6538 in an in vitro competition assay [84]. Furthermore, β-glucan metabolism significantly increased the concentration of lactic acid, a natural moisturizing factor of the stratum corneum skin barrier, which contributed to skin hydration with a slightly acidic pH.

Li et al. investigated the impacts of four prebiotic oligosaccharides (FOSs, GOSs, IMOs, and inulin) on the in vitro growth of a skin commensal *S. epidermidis* CCSM0287 (a potential skin probiotic) [23]. Their results showed that all four oligosaccharides (2% (*w*/*v*) in final media) supported the growth of *S. epidermidis* CCSM0424, with FOSs being the most effective. Short-chain fatty acid (SCFA) analysis indicated that the fermentation of oligosaccharides by *S. epidermidis* CCSM0287 produced acetic acid and isovaleric acid, which was different from the metabolites from gut bacteria. In addition, the post-fermentation supernatant (postbiotics) from 2% FOSs significantly inhibited *S. aureus* CCSM0424 biofilm formation. Their study proved that FOSs are a potential skin prebiotic that significantly promotes the growth of *S. epidermidis* and inhibits *S. aureus* biofilm formation, thus exerting the probiotic effects of *S. epidermidis.*

Fourniere et al. studied the production of oligosaccharides from *Ulva* sp. (green alga) and their effects on the skin bacteria *S. epidermidis*, *S. aureus*, and *C. acnes* [87]. The oligosaccharides produced from the enzymatic extraction and depolymerization of *Ulva* sp. (Ulvan) consisted of 24.4–30.4% carbohydrates (44.9–55.4% rhamnose and 7.5–11.0% glucuronic acid) and 21.6–30.8% uronic acid, with a molecular weight of 8 kDa and 1.5 kDa, respectively. The Ulvan-derived oligosaccharides at a concentration of 1000 μg/mL did not affect the bacterial growth of *S. aureus* MFP03 and *S. epidermidis* MFP04 in terms of generation time, but they were able to modify their biofilm structures in vitro. In addition, the Ulvan-derived oligosaccharides did not significantly change the cytotoxic potential of *S. epidermidis* and *S. aureus* towards HaCaT keratinocytes. Also, the inflammatory potential of both acneic and non-acneic *C. acnes* strains on HaCaT keratinocytes was decreased up to 39.8% by the treatment with the oligosaccharides. The results suggested the potential of Ulvan-derived oligosaccharides for dermo-cosmetic applications.

**Figure 5:**
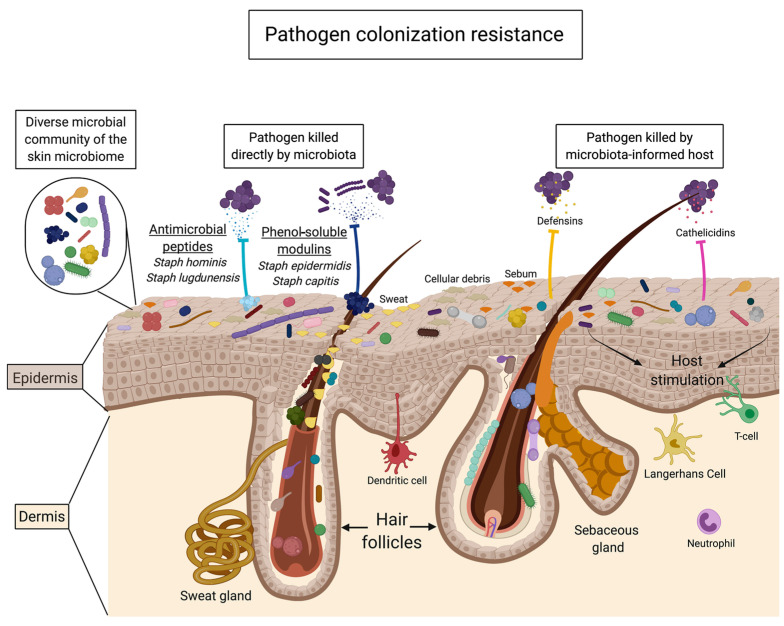
Mechanisms of pathogen colonization resistance mediated by the skin microbiota (reproduced with permission from [3]).

From the above studies, several mechanisms are suggested to explain the effects of oligosaccharides on the promotion of skin commensals and/or inhibition of pathogens. First, prebiotic oligosaccharides act as a direct carbon source for the growth of skin commensals, rather than pathogens, and produce fermentation metabolites, such as lactic acid and SCFAs, to modulate skin pH for a favorable microenvironment [23,84]. Alternatively, skin microbes that metabolize prebiotic oligosaccharides can also provide substrates for the growth of non-metabolizing microbes, thus reinforcing commensal networks through cross-feeding and preventing pathogen overgrowth [3,98]. In terms of pathogen colonization resistance, two pathways have been proposed for the metabolism of prebiotic oligosaccharides (Figure 5): (1) skin commensals secrete antimicrobial metabolites, such as bacteriocins, and phenol soluble modulins, to directly suppress pathogens or alter the virulence of pathogens and (2) skin commensals signal to the host to mount a protective immune response to invading pathogens [3].

### 3.2. Anti-Melanogenesis (HMOs, GOSs, AOSs)

**Figure 6:**
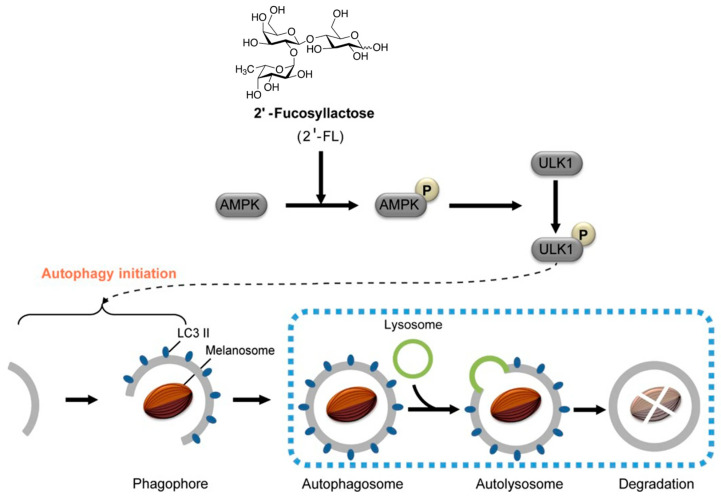
Human milk oligosaccharide 2′-fucosyllactose (2′-FL) promotes melanin degradation via the autophagic AMPK–ULK1 signaling axis. 2′-FL activates AMPK, which, in turn, phosphorylates ULK1 at Ser555 and inhibits Ser757-mediated inactivation, with consequent autophagy initiation (reproduced with permission from [89]).

Kwak et al. reported that 2′-fucosyllactose (2′-FL), a major component in HMOs, was considered as a potential skin whitening cosmetic material with anti-melanogenic effects [88,89]. In vitro assays on MNT-1 cells and human-derived melanocytes showed a 40% reduction in melanin production with 2′-FL treatment (no cytotoxicity at 20 g/L) [88]. The anti-melanogenic effects of 2′-FL are mediated through the AMPK-ULK1 autophagy pathway (Figure 6). 2′-FL activates AMPK, which phosphorylates ULK1 at Ser555, initiating autophagy while inhibiting mTORC1. This triggers the formation of autophagosomes, marked by light chain 3 (LC3)-II conversion, which engulf melanosomes for lysosomal degradation. Concurrently, 2′-FL downregulates melanogenic enzymes (tyrosinase and TYRP1) and, thus, suppresses melanin production [89]. Studies confirm that this pathway—validated by AMPK inhibition experiments—leads to ~40% melanin reduction in melanocytes without cytotoxicity, positioning HMOs as promising natural agents for treating hyperpigmentation in cosmetics [88,89].

Suh et al. reported the inhibitory effects of GOSs on skin pigmentation through in vitro and clinical trials [29]. In vitro cell viability tests suggested that there was no cytotoxicity on B16F10 cells with GOS concentrations of 70 mg/mL or less (GOS purity of 74.9%). Melanin production in B16F10 cells was significantly inhibited by 14 mg/mL GOS (*p* < 0.05), and treatment with 70 mg/mL GOS decreased about 25% of the melanin production. In addition, 14–35 mg/mL GOS showed a positive effect on UVB-irradiated HaCaT keratinocytes, but higher concentrations (70 mg/mL) inhibited cell viability, possibly because of osmotic pressure (rather than GOS toxicity). Clinical trials suggested that after a 12-week GOS treatment (1 g, twice per day) on healthy women (40–60 yrs, N = 84), the melanin index and erythema index both significantly decreased (*p* < 0.05), suggesting the potential role of GOSs as a nutritional approach for the inhibition of skin pigmentation.

Kim et al. investigated the enzymatic production of 3,6-anyhydro-_L_-galactose (AHG) from agarose (red algae) and its skin whitening properties [90]. An in vitro skin whitening assay indicated that 100 μg/mL AHG was able to significantly suppress melanin production in murine B16F10 melanoma cells, and AHG up to 200 μg/mL did not show significant cytotoxicity. In addition, AHG showed strong anti-inflammatory activity in suppressing in vitro nitrite production. Later, the same group systematically investigated the skin whitening activity of AHG and AHG-containing oligosaccharides (agaro-oligosaccharides (AOSs), neoagaro-oligosaccharides (NAOSs)) via in vitro cell assays [91]. It was found that AHG, AOSs, and NAOSs at 12.5/25/50 μg/mL did not exhibit cytotoxicity toward murine B16 melanoma cells or human epidermal melanocytes (HEMs). Moreover, AHG significantly reduced the intracellular melanin content in B16 cells and HEMs and NAOSs (DP 4 and DP 6) exerted a moderate anti-melanogenic effect, while NAOSs (DP 2) and AOSs (DP 3, DP 5, and DP 7) did not reduce melanin production. The authors claimed that AHG is responsible for the anti-melanogenic activity in AOSs. The structural differences explained their difference in skin whitening properties, i.e., the non-reducing end of NAOSs contains AHG, while that of AOSs contains galactose.

Recently, Aisa et al. reported that a novel oligosaccharide compound (referred to as ACO-II-1), extracted from almond, with a structure of β-D-Fru*f*-(1→2)-β-D-Fru-(1→6)-α-D-Glc*p*-(1→1)-α-D-Glc*p*, showed strong antioxidant activity and an inhibition effect on melanogenesis in forskolin (FSK)-induced B16F10 melanoma cells [99]. ACO-II-1 was able to reduce intracellular melanin content and tyrosinase activity, with a reduced expression of tyrosinase, TRP-1, TRP-2, and microphthalmia-associated transcription factor (MITF). An investigation into the mechanism showed that the anti-melanogenic properties of ACO-II-1 were mediated through mitogen-activated protein kinases (MAPKs) and β-catenin signaling pathways.

### 3.3. Antioxidant and Anti-Inflammation Activities (AOSs, GlcOSs, FOSs, GOSs, XOSs)

In a recent study by Lee et al., a mixture (referred to as AO13) of 3,6-anyhydro-_L_-galactose (AHG) and agaro-oligosaccharides (AOSs, DP 3) exerted strong antioxidant activity in human dermal fibroblasts (HDFs) in vitro [92]. Specifically, AO13 exhibited no cytotoxicity in HDFs and promoted cell proliferation. In addition, AO13 showed higher reactive oxygen species (ROS)-scavenging activity than the neoagaro-oligosaccharides (NAOSs, DP 2 and DP 4) in H_2_O_2_-induced HDFs in vitro, mainly because of the structural difference, i.e., AOSs contain galactose at the non-reducing end, whereas NAOSs contain AHG. Since ROS promote skin aging by driving extracellular matrix degradation in the dermis, the authors suggested that odd-numbered AOSs might help prevent skin aging by protecting against oxidative stress, thus exerting antioxidant activity.

Park et al. reported the synthesis, prebiotic effect, and anti-inflammatory properties of α-gluco-oligosaccharides (α-GlcOSs) produced by *Leuconostoc lactis* SBC001 [93]. Glucansucrases produced by *Leuconostoc lactis* SBC001 catalyzed the transglycosylation reaction of maltose and sucrose, which produced α-GlcOSs consisting of 24% α-(1→4) and 76% α-(1→6) linkages with an average molecular weight of 1137 Da (average DP around 7). The resultant α-GlcOSs showed anti-inflammatory activity towards lipopolysaccharide-stimulated RAW 265.7 macrophage cells in vitro. Specifically, α-GlcOS treatment decreased the production of nitric oxide by suppressing the expression of nitric oxide synthase. In addition, the expression levels of tumor necrosis factor-α and inflammatory cytokines (IL-1β, IL-6, and IL-10) were suppressed. Also, the nuclear factor-kappa B signaling pathway was inhibited by α-GlcOS treatment. Furthermore, it was predicted that the anti-inflammatory properties of α-GlcOSs were attributed to the reduced inflammatory response by suppressing the inflammatory-associated genes and cytokines in the NF-κB signaling pathway and the MAPK signaling pathway. On the other hand, α-GlcOSs promoted the in vitro growth of six bacterial (potential probiotic) and yeast strains, exerting their prebiotic potential.

Chang et al. prepared pectic oligosaccharides (POSs) of different molecular weights via hydrolysis of okra pectin (112.31 kDa) by the Fenton reaction using varied concentrations of FeSO_4_. They found that the smallest POSs (1.79 kDa) showed the highest prebiotic, antioxidant, and anti-inflammatory activities [94]. Specifically, the POSs promoted the in vitro growth of two probiotic strains (*Lacticaseibacillus rhamnosus* ATCC 7469 and *Bifidobacterium longum* ATCC 15707) with increased SCFA levels. Compared to the original pectin, the POSs with lower molecular weights showed stronger antioxidant activities by scavenging DPPH and ABTS radicals. Moreover, the POSs inhibited LPS-induced nitrite and inflammatory cytokines (interleukin (IL) IL-1β and IL-6) and also inducible nitric oxide synthase (iNOS)/NF-κB signaling pathways in RAW 264.7 cells.

**Figure 7:**
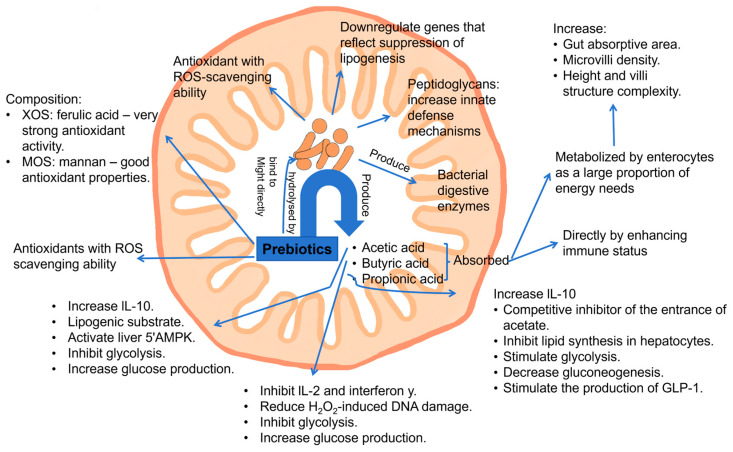
General action mechanisms of prebiotics (oligosaccharides and polysaccharides) of antioxidant and anti-inflammatory effects (reproduced with permission from [104]). Notes: XOSs: xylo-oligosaccharides; MOSs: mannan oligosaccharides; ROS: reactive oxygen species; IL-10: interleukin-10; GLP-1: glucagon-like peptide-1.

GOSs, FOSs, XOSs, chitio-oligosaccharides, and other polysaccharides, such as fructans, arabinoxylans, and *β*-glucans, were reported to have strong antioxidant activity and to act as reactive oxygen species (ROS) scavengers by scavenging free radicals, such as the superoxide anion (O_2_^•−^), hydroxyl radical (^•^OH), and nonradical molecules, including hydrogen peroxide (H_2_O_2_) and singlet oxygen (^1^O_2_) [100]. The mechanism (Figure 7) was suggested to be that oligosaccharide metabolism and production of SCFAs are able to induce the expression of crucial antioxidant enzymes, such as glutathione S-transferases (GSTs), and reduce the generation of oxidative products during digestion and colonic fermentation [101,102]. GSTs are a family of enzymes that catalyze the conjugation of glutathione to various substrates, thereby neutralizing ROS) and other oxidative products. By enhancing the expression of GSTs, oligosaccharides effectively reduce the oxidative burden within the body, protecting cells and tissues from oxidative damage. Furthermore, in vivo studies have highlighted a potential synergistic effect between prebiotic oligosaccharides and polyphenols [103]. When oligosaccharides, such as FOSs and GOSs, are metabolized in the large intestine, the resulting SCFAs may enhance the bioavailability and efficacy of polyphenols. This synergistic interaction provides an early defense against oxidative stress, as the combined action of SCFAs and polyphenols can more effectively neutralize ROS and other free radicals compared to either component alone.

### 3.4. Wound Healing (GOSs, Thiolated Oligosaccharides)

Silvagno et al. conducted in vitro assays using human keratinocyte cell lines and reported the beneficial effect of GOSs (prepared from whey permeate) on wound healing and skin health due to their direct activity on keratinocyte functions [30]. GOS treatment (3%, 7 days) stimulated a beneficial reversible inflammatory response (IL-8 upregulated by nuclear factor kappa B signaling) in HaCaT cells and promoted cell migration and E-cadherin loss without epithelial–mesenchymal transition. In addition, GOS treatment led to enhanced mitochondrial function associated with the translocation of the Forkhead Box O1 transcription factor. GOSs also triggered an increased expression of cell differentiation markers that contribute to wound healing processes. These results suggested the potential topical application of GOSs for wound healing purposes.

Santos et al. found that dietary supplementation of inulin and GOSs can improve colonic healing and surgical recovery in mice experiments with a surgical colonic anastomosis [95]. Mice were fed a diet containing inulin, GOSs, or a non-fermentable dietary fiber cellulose (all 10 wt/wt%) for two weeks before undergoing colonic anastomotic surgery. It was found that oligosaccharides improved the general health of operated mice, based on increased body weight, and induced the thickening of the colonic wall. In addition, inulin/GOS supplementation resulted in significantly higher levels of SCFAs, such as butyrate, lowering the incidence of anastomotic leaks. The improved anastomotic healing was mostly attributed to an enhanced re-epithelialization of the wound, indicated by increased epithelial proliferation (indicated by mucosal layer). In addition, inulin and GOS diets resulted in elevated collagen levels in peri-anastomotic tissue and an inhibitory effect on collagenases, such as matrix metalloproteinases, responsible for collagen degradation.

**Figure 8:**
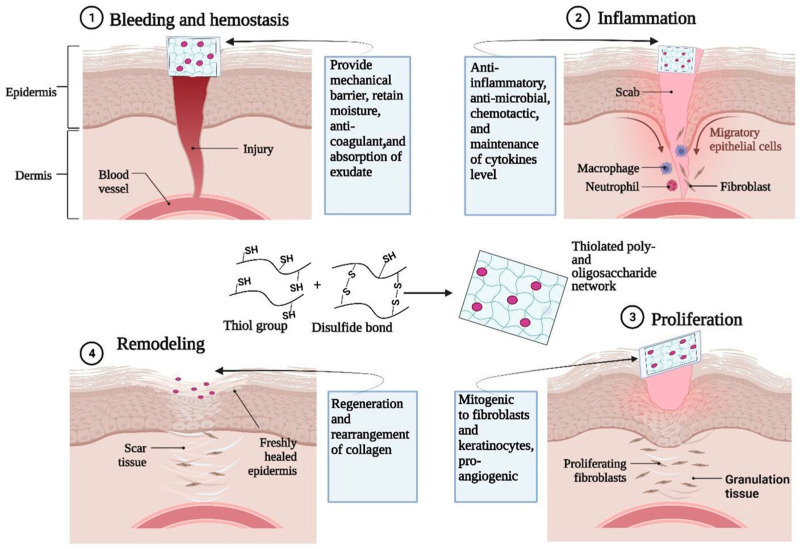
Role of thiolated poly- and oligosaccharide-based (e.g., oligosaccharides from chitosan, hyaluronic acid) hydrogels for different stages of wound healing applications (reproduced with permission from [96]).

Schnürch et al. found that thiolated poly- and oligosaccharide-based hydrogels are highly advantageous for tissue engineering and wound healing, such as thiolated chitosan and thiolated hyaluronic acid (Glycosil) [96]. Thiolated poly- and oligosaccharide-based hydrogels have good biocompatibility, biodegradability, and nontoxicity and perform well in the role of wound dressings with unique features, such as in situ gelling, cell adhesion (bioadhesion), drug release control, enzyme inhibition, and metal binding properties (mitigate toxic effects). These features are primarily attributed to their capability to form disulfide bonds with each other and/or endogenous proteins. Figure 8 illustrates the functional roles of thiolated poly- and oligosaccharide-based hydrogels in the four key stages of the wound healing process. The mechanism underlying the effectiveness of thiolated poly- and oligosaccharide-based hydrogels as wound dressings can be attributed to their capacity to absorb and retain wound exudates, thereby creating a conducive environment for critical biological events, such as fibroblast proliferation and keratinocyte migration.

## 4. In Vivo Studies of Prebiotic Oligosaccharides on the Skin Microbiome and Related Skin Health Effects

This section summarizes the in vivo animal and human trials that have investigated the effects of prebiotic oligosaccharides on the skin microbiome and related skin health benefits, as detailed in Table 3. By examining oral and topical administration methods, this section elucidates how prebiotic oligosaccharides modulate skin and gut microbiota to confer benefits, such as alleviation of atopic dermatitis, acne attenuation, enhanced skin hydration, and anti-aging effects. The discussion also addresses mechanistic insights, clinical outcomes, and the role of microbial metabolites (e.g., SCFAs) in mediating these effects, offering a holistic perspective on their therapeutic and cosmetic potential.

### 4.1. Alleviation or Prevention of Atopic Dermatitis (HMOs, FOSs, GOSs, AOSs)

**Figure 9:**
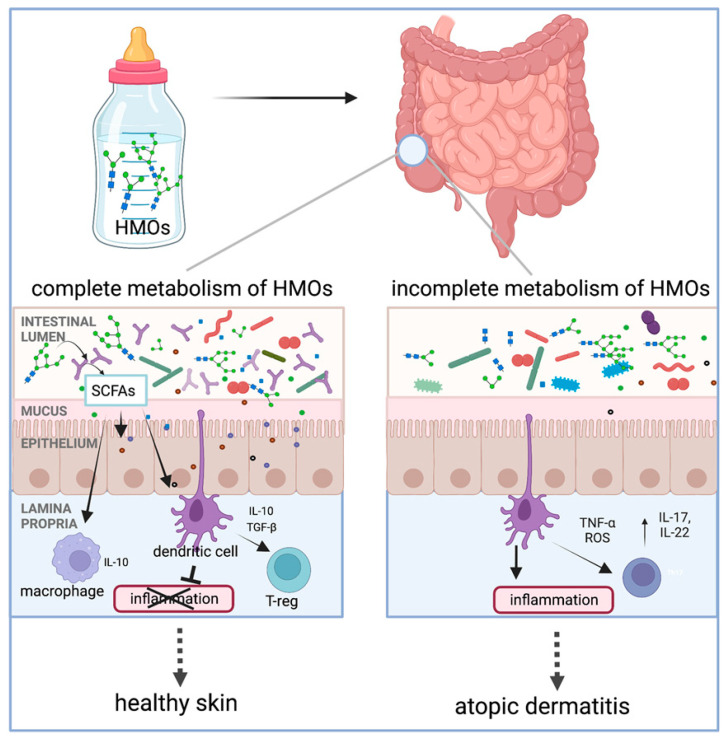
Metabolism of human milk oligosaccharide (HMO)-induced immunological effects and protection of skin from atopic dermatitis development (reproduced with permission from [17]). Notes: SCFAs: short-chain fatty acids; IL: interleukin; TGF-β: transforming growth factor-β; T-reg: regulatory T cells; TNF-α: tumor necrosis factor α; ROS: reactive oxygen species.

HMOs play an important role in establishing the infant gut microbiota and have immunomodulatory effects on the infant gut system, which have been gradually examined with preventive effects against atopic dermatitis (AD)/eczema in infants [119,120]. Several studies and meta-analyses have reported a protective role of HMOs against AD when infants are fed human milk, particularly during the first 4 months of life [17,121]. The action mechanism of HMOs was summarized in a recent review by Ceraj et al. [17], which is illustrated in Figure 9. Specifically, HMOs act as food for infant gut microorganisms, such as *B. infantis*, *Bacteroides* spp., and others, producing metabolites like SCFAs upon metabolism. Then, SCFAs can modulate the immune response by interacting with dendritic cells that extend dendrites into the gut lumen or by crossing the epithelial barrier to directly interact with immune cells in the lamina propria. As a result, interleukins, such as IL-10, are released by dendritic cells and function in the regulation of inflammation. The authors found that infants with microorganisms that have the complete set of HMO metabolizing genes are protected from the development of AD/eczema and/or experience reduced severity of AD/eczema. In addition, Thanabalu et al. reported that SCFAs from the metabolism of prebiotic oligosaccharides affect peripheral inflammation by locally promoting iTreg development in the intestine that then migrate to target skin, where the Th2 cell response was suppressed, resulting in a protective effect against atopic dermatitis [105].

Tang et al. reported that dietary GOSs ameliorated atopic dermatitis (AD)-like skin inflammation and behavioral deficits (anxiety, depression) by modulating the gut microbiota–brain–skin axis [18]. GOS treatment (purity >95%, 0.5 or 1.5 g/kg) markedly relieved skin inflammation by decreasing the production of inflammatory cytokines IgE, IL-4, IL-13, IFN-γ, and TNF-α and regulating PPAR-γ/NF-κB signaling in 2,4-dinitrofluorobenzene (DNFB)-induced AD mice (N = 40). Furthermore, GOS treatment significantly improved anxiety- and depressive-like symptoms by normalizing the neurotransmitter levels of 5-HT, DA, NE, and CORT in the brain. The underlying mechanisms were that GOSs restructured the gut microbiota and specifically promoted *Lactobacillus* and *Alloprevotella*, which increase fecal SCFAs, particularly acetate and butyrate. A marked correlation between the altered intestinal microbiota/SCFAs, AD-associated symptoms, and comorbid behavioral phenotypes (anxiety, depression) was substantiated by Pearson correlation analysis.

Kim et al. investigated the mechanism through which kestose-enriched FOSs alleviated AD and compared it to the effects of normal FOSs [106]. The oral administration (7.3 mg powder/day or 4.9 mg syrup/day) of kestose-enriched FOSs (>85% kestose) in ovalbumin-sensitized Balb/c mice was found to increase fecal propionic and butyric acid levels to a greater extent than that of normal FOSs (33% kestose). This was the result of FOS-modulated gut microbiota, with an increasing abundance of *Lactobacillus* and *Alistipes* and a decreased abundance of *Prevotella*. Moreover, kestose-enriched FOSs significantly reduced the serum levels of Th2 (IL-4, IL-5, IL-13, TARC, and eotaxin) and Treg cytokines (IL-10 and IL-1β) while increasing Th1 cytokines (TNF-α, IFN-γ, and IL-12). This indicated that the allergic inflammation of AD was suppressed by kestose-enriched FOSs through their immunological recovery of the Th1/Th2 balance.

Ren et al. reported that the alginate oligosaccharides (AOSs) of different structures mediated the gut–skin axis balance and prevented skin aging in mouse models [107]. Oral gavage of all AOSs (10 mg/kg) significantly upregulated skin aging phenotypes (increased stratum corneum water content, skin elasticity, epidermal thickness, collagen thickness, increased DEJ tortuosity, and reduced fibrotic area) in aging mice. Among the three types of AOSs, MAOSs (mannuronate oligosaccharides, 6.44 kDa, mannuronate/guluronate ratio 12.56) markedly mediated the colonic butyrate-HIF-1α axis homeostasis, which promoted the entry of butyrate into the skin, upregulated the mitophagy level, and ultimately improved skin aging via the HDAC3/PHD/HIF-1α/mitophagy loop in mice.

### 4.2. Prevention of Allergies (HMOs, GOSs, FOSs)

**Figure 10:**
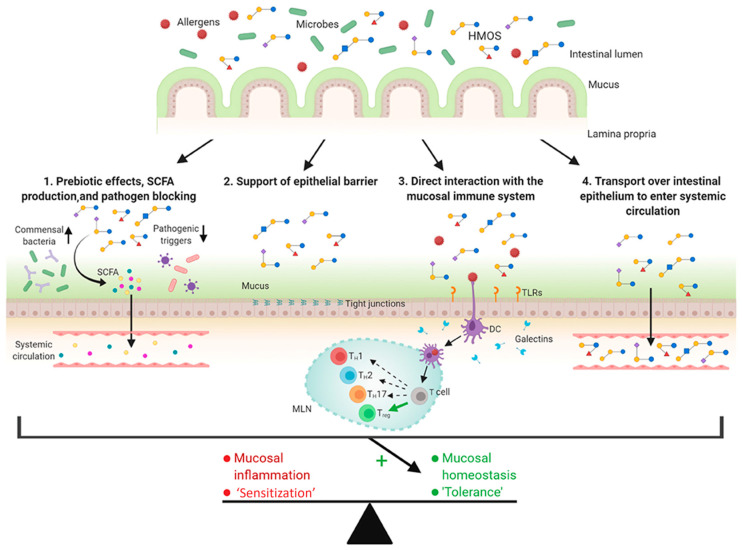
Possible mechanisms of human milk oligosaccharides (HMOs) in the prevention of allergic diseases (reproduced with permission from [122]). Notes: SCFAs: short-chain fatty acids; DCs: dendritic cells; TLRs: toll-like receptors.

Allergic diseases, such as respiratory allergies, food allergies, and atopic dermatitis, have shown increasing prevalence, particularly among children and infants, which has triggered interest in dietary intervention in early life to modulate gut and immune maturation. HMOs have been shown to exhibit effects in the modulation of the gut microbiome [33,52] and mucosal immune response, which play a potential role in the prevention of allergic diseases [17]. Zuurveld et al. summarized the possible functions of HMOs related to the protection of allergic diseases at a young age (Figure 10) [122]. First, HMOs act as prebiotics by promoting commensal bacteria growth and inhibiting pathogen adhesion to the intestinal epithelium. HMO fermentation to SCFAs strengthens epithelial barrier function and modulates immune responses locally and systemically. Second, HMOs boost mucus secretion and tight junction integrity, reinforcing the physical barrier between the intestinal epithelium and the gut content. Thirdly, HMOs directly regulate immune function by influencing the dendritic cell response. Finally, a small fraction of HMOs transported over the intestinal epithelium exert immunomodulatory effects beyond the gut. These HMO-mediated mechanisms may promote immune tolerance and, therefore, potentially prevent allergic diseases.

Bodinier et al. reviewed the preventive effects and mechanisms of prebiotic oligosaccharides, such as GOSs, FOSs, and other emerging prebiotic oligosaccharides (e.g., XOSs, IMOs), in allergies [123]. The authors summarized two distinct mechanisms (direct and indirect effects) by which prebiotic oligosaccharides can influence host health. Indirect effects were attributed to the SCFAs produced from the fermentation of prebiotic oligosaccharides by gut bacteria, which impact epithelial cells and immune cells and thereby potentially influence the prevention of allergies. On the other hand, the direct function of prebiotic oligosaccharides on the epithelial cells of the lung, skin, gut, and immune system was observed. For example, prebiotic FOSs/GOSs suppressed the excessive production of TSLP, substance P, IL-4, IL-10, and TNF-α, which contributed to the prevention of keratin depletion, an improvement in epidermal biophysical properties, the restoration of skin sebum levels, and a reduced bacterial infection risk. Additionally, prebiotics promoted immune tolerance by increasing CD4+ Foxp3+ Treg cells in skin-draining lymph nodes, inhibiting germline class-switching and IgE production.

### 4.3. Attenuation of Acne Vulgaris (COSs, FOSs, GOSs)

Acne vulgaris is a common inflammatory skin disease that is closely related to the skin microbiome [124,125]. *Cutibacterium acnes* (*C. acnes*, formerly *Proprionibacterium acnes*), a Gram-positive anaerobic bacterium, has long been considered as a skin commensal and has emerged as an opportunistic pathogen that contributes to the inflammatory phase of acne [126]. Current acne treatments focus on the use of doxycycline, benzoyl peroxide, isotretinoin, etc. [125], and prebiotic oligosaccharides have been studied as an emerging therapy for acne by restoring the microbial equilibrium or selectively targeting pathogenic strains. For example, Kim et al. reported the antibacterial activities of chitosan oligosaccharides (oligochitosan) against acne-related bacteria, particularly *C. acnes* [85]. It was found that 10 kDa oligochitosan presented the highest antimicrobial effect with minimum inhibitory concentration values of 32–64 μg/mL on *C. acnes*. In addition, an antibacterial synergy effect was observed when combining oligochitosan with tetracycline or erythromycin, indicated by a median ΣFIC (fractional inhibitory concentration) range of 0.02–0.56. The mechanism was presumably attributed to the fact that oligochitosan can be stacked over the microbial cell surface, blocking nutrients, or bind to DNA, thus inhibiting transcription or the permeability of the microbial cell wall.

Furthermore, Ruxton et al. summarized the effects of a novel seaweed oligosaccharide–zinc complex (SOZC) on reducing the symptoms of acne vulgaris [110]. The seaweed oligosaccharides were prepared by the extraction of polysaccharides from brown seaweed (*Laminaria digitata*), followed by enzymatic depolymerization and, finally, chelating with ZnSO_4_ to form a zinc complex (Phycosaccharide^®^ AC, The Mentholatum Company, East Kilbride, United Kingdom). In vitro studies proved that 5.6% SOZC reduced *C. acnes* (contributing to the inflammatory phase of acne) counts by 74%, and SOZC was found to have soothing effects, indicated by increased levels of interleukin 1 alpha by 11.1%. In addition, double-blind human trials suggested that treatment with 5% SOZC significantly improved acne vulgaris symptoms in 14 days, with reduced sensitivity and improved skin healing. The likely mechanisms of action may include antibacterial, antioxidant, and anti-inflammatory effects related to brown seaweed, and a synergistic effect might be attributed to the zinc complex.

A recent human trial study was conducted to investigate the effects of oral supplementation with FOSs and GOSs in women with severe acne [108]. The results suggested that oral FOSs (100 mg) and GOSs (500 mg) for 3 months significantly reduced fasting blood glucose levels (*p* = 0.02), total cholesterol levels (*p* = 0.018), and triglycerides (*p* = 0.05), but there was a nonsignificant decrease in insulin and C-peptide plasma. Overall, oral FOSs and GOSs led to positive effects on glycemic and lipid metabolic parameters in women with adult acne. Additionally, FOS and GOS supplements may reduce systemic inflammation, oxidative stress, and insulin resistance, all of which contribute to acne pathogenesis [124].

### 4.4. Skin Hydration, Anti-Dryness, and Anti-Itching (GlcOSs, GOSs, FOSs, Colloidal Oat)

Prebiotic oligosaccharides are able to improve water retention on the skin and reduce dryness [28,112]. For example, Suh et al. found that the oral administration of prebiotic GOSs and probiotic *B. longum* was able to prevent transepidermal water loss and reduce erythema formation in hairless mice studies [111]. Berardesca et al. investigated the efficiency of α-gluco-oligosaccharides (α-GlcOSs) and collagen tripeptide F in controlling symptoms in females with sensitive atopic skin (N = 40, 30–59 yrs) [113]. A 4-week treatment (α-GlcOSs in a lotion) was found to significantly improve skin barrier properties, including skin hydration, roughness, skin pH, and transepidermal water loss (TEWL). A clinical assessment indicated reduced dryness, desquamation, and skin irritability after treatment, which helped improve the symptoms of sensitive skin. No significant difference was observed in the number of *Staphylococcus* colonies after the treatment.

Kano et al. conducted a double-blind, placebo-controlled, randomized trial and found that a daily intake (100 mL, 4 weeks) of Yakult fermented milk containing prebiotic GOSs and probiotic *B. breve* (YIT 12272) improved skin hydration in healthy adult women (N = 40) [28]. The hydration levels and cathepsin L-like activity (related to keratinocyte differentiation) in the stratum corneum turned out to be significantly higher in the active group, while the serum and urine phenol levels (e.g., phenol, *p*-cresol, regarded as bioactive toxins) were significantly lower.

Kim et al. reported that the oral administration of 4.25 g FOS (1-kestose) significantly alleviated itching and sleep disturbance symptoms and improved the skin barrier function in children (N = 48, 2–17 years) with atopic dermatitis through a randomized, double-blind, placebo-controlled trial for 12 weeks [19]. Skin microbiome composition analysis revealed that FOS treatment decreased the abundance of *Lachnospiraceae* and increased the abundance of *Prevotella* and *Rothia* in AD skin, while the control group (maltose) resulted in a decreased abundance of *Methylobacterium*. Furthermore, FOSs enhanced the skin barrier function by altering the proportion of linoleic acid (18:2) esterified omega-hydroxy-ceramides (EOS-CERs), with significantly decreased levels of amide-linked short-chain fatty acids (C28 and C30). The mechanisms underlying the positive effects of FOSs were attributed to increased expression of *ELOVL3* (a key enzyme in the elongation of C16-C22 fatty acids) and FLG (a key epidermal barrier protein that maintains skin barrier function).

Southall and colleagues reported that the anti-inflammatory activities of colloidal oatmeal (65–85% starch, 15–20% protein, 3–11% lipid, 5% fiber, and 5% β-glucan) and its protective effect for dry, irritated skin [114]. In vivo clinical trials showed that the colloidal oatmeal skin protectant lotion applied on healthy females (N = 29) with itchy, dry skin on their lower legs led to significant improvement in skin dryness, scaling, roughness, and itch intensity during a 2-week application (twice a day). In vitro assays revealed that the extracts of colloidal oatmeal significantly inhibited inflammatory mediators (pro-inflammatory cytokines IL-8, NF-κB luciferase promoter levels) associated with skin inflammation and, in turn, enhanced the skin benefits of colloidal oatmeal for dry, irritated, and eczematous skin. In a similar study, Liu-Walsh et al. reported that the 6-week use of a daily moisturizer containing 1% colloidal oat significantly promoted the growth of *S. epidermidis* ATCC 12228 and increased the concentration of lactic acid (from bacterial fermentation) on the skin of female subjects with skin dryness issues [84]. Lactic acid is considered as a natural moisturizing factor of stratum corneum, which helps to maintain skin hydration and a slightly acidic skin pH [127].

### 4.5. Anti-Aging and Photoprotection (GlcOSs, XOSs, GOSs, COSs)

Zahr et al. investigated the effects of a moisturizer formulated with prebiotic α-glucan oligosaccharides and postbiotics (*Pseudoalteromonas* ferment extract) on 25 female subjects (35–65 yrs, mean age 54 ± 6) with Fitzpatrick skin types I–VI, moderate crow’s feet wrinkles, and global face photodamage [26]. A 28-day treatment (twice daily application) significantly increased the microbial facial diversity (α-diversity) and shifted the microbiota composition (e.g., reduced *Cutibacterium acnes* abundance). In addition, a clinical study suggested a 10.3% average improvement for 85.7% of subjects in tactile firmness (** *p* < 0.001), a 12.6% average improvement in overall photodamage (*** *p* < 0.001), and a 25.9% average improvement in full face radiance (** *p* < 0.001). The results suggested that the multifunctional moisturizer specifically targeted the dermal–epidermal junction (DEJ) and skin microbiome to improve the diversity and balance of the facial microbiome at the species level while providing anti-aging benefits.

Zhao et al. found that a dual intervention (oral and topical) of xylo-oligosaccharides in human females (N = 77) attenuated facial cutaneous aging, with decreased facial *Cutibacterium* abundance and enriched intestinal *Bifidobacterium* [21]. The mechanism was predicted to be the decreased fructose-1-phosphate kinase (encoded by K02770) involved in de novo lipid synthesis, thus inhibiting lipophilic *Cutibacterium* with reduced facial pores.

Suh et al. found that the oral treatment of prebiotic galacto-oligosaccharides (100 mg, 12 weeks) and/or probiotic *Bifidobacterium longum* (*B. longum*, 10^9^ CFU) protected against UV-induced skin damage (wrinkle formation, skin thickening, and water loss) in hairless mice [111]. The effect was likely attributed to increased levels of gene (TIMP-1 and Col1) expression related to collagen synthesis and degradation. In addition, the treatment was found to increase the water-holding capacity of the skin, prevent transepidermal water loss, and reduce erythema formation (16.8%) of mice skin after exposure to UV. Similarly, Kim et al. reported the protective effects of dietary GOSs and *B. longum* against UVB-induced photo-aging and also anti-inflammatory and antioxidative effects [116].

Hong et al. reported the photoprotective effect of dietary GOSs (mainly DP 2–4 with β-(1→4) and β-(1→6) glycosidic bonds) in hairless mice by regulating the mitogen-activated protein kinase (MAPK) signaling pathway [115]. The 8-week oral administration of GOSs (200 mg/kg) increased TEWL, decreased the water-holding capacity, and also significantly reduced the wrinkle area and mean wrinkle length in hairless mice exposed to UVB. In addition, GOS ingestion significantly reduced the levels of inflammatory cytokines (IL-6, IL-12, TNF-α) induced by UVB irradiation. Moreover, GOS intake significantly prevented the UVB-induced MAPK phosphorylation of JNK, p38, and ERK kinases that contributed to skin aging. Similarly, Jung et al. reported that the oral administration of collagen tripeptide and GOS mixtures (3:1, 1:1, and 1:3) showed a synergistic inhibitory effect on photo-aging in UVB-exposed hairless mice through changes in gene expression, cytokine levels, and intestinal microbiota composition [117].

Kong et al. investigated the potential preventive effects of chitosan oligosaccharides (COSs, average Mw = 1000 Da, degree of deacetylation ≥90%, water-soluble) in UV-irradiated hairless mice skin [27]. A 10-week topical application of COSs at a concentration of 50/100/200 mg/mL was found to effectively alleviate the UV-induced macroscopic and histopathological damage of mice skin by mitigating the disrupted collagen fibers and increasing the content of type I collagen and total collagen. Moreover, COSs inhibited the production of pro-inflammatory cytokines (e.g., TNF-α, IL-1β, IL-6), significantly enhanced the activities of antioxidant enzymes (SOD, GSH-P_x_, CAT), and increased the content of skin hydroxyproline (positively correlated to dermal collagen) and moisture. The authors therefore concluded that COSs were a promising agent for preventing skin photo-aging and promoting skin health.

Jiang et al. studied the preparation of succinyl-chitosan oligosaccharides (SU-COSs) and evaluated their protective effects against UVB-induced skin damage both in vitro and in vivo [22]. SU-COSs (6400 Da, substitution degree of 69.26%) showed good biocompatibility with L-929 cells, erythrocytes, and subcutaneous skin in an in vitro hemolysis assay. SU-COSs effectively promoted the 2D and 3D migration of human epidermal HaCaT cells in a scratch migration assay. In vitro SU-COS treatment on UVB-irradiated HaCaT cells enhanced cell viability (increased survival ratio by <11%), maintained cytoskeletal morphology, and stabilized the cell cycle while concurrently reducing intracellular reactive oxygen species (ROS) formation. In vivo studies demonstrated that the SU-COS intervention (200/400 mg/kg) showed a protective effect on UVB-irradiated mice skin by reducing skin erythema and transepidermal water loss, relieving crusting. Furthermore, SU-COSs also maintained the integrity of the epidermis and skin thickness, as well as the stability of collagen fibers. The protective effects of SU-COSs were correlated with the modulation of gene and protein expression associated with the cellular cycle, collagen synthesis, extracellular matrix degradation, antioxidant defense, and anti-inflammation properties. Taken together, SU-COSs were identified as a potential treatment for repairing UVB-induced photodamage.

Zhao et al. reported a protective effect of *Panax ginseng* oligosaccharides (GSOs) against skin barrier damage induced by UVB [118]. The GSOs (1000 Da) were composed of seven monosaccharide units (62.2% glucose, 9.0% galacturonic acid, 8.4% galactose, 5.8% arabinose, 5.5% glucuronic acid, 5.1% mannose, 3.9% rhamnose) linked by β-bonds. Topical treatment (0.2/1.0/2.0 mg/cm^2^/day) of GOSs on the dorsal skin of BALB/c hairless mice reduced UVB-induced epidermal thickening and moisture loss. Specifically, the GSOs attenuated UVB-induced skin dryness by increasing the expression of related proteins (CE and AQP3). In addition, GSO treatment reduced desquamation both in vivo and in vitro (using HaCaT cells) by enhancing the expression of desquamation-related proteins (SPINK5, KLK5, KLK7, and DSG1).

### 4.6. Mechanism of Action of Oligosaccharides

Table 4 summarizes the key pathways through which different oligosaccharides exert their skin health benefits, highlighting the importance of the administration route (oral vs. topical) in determining their mechanisms of action. Orally administered oligosaccharides, such as HMOs, GOSs, and FOSs, primarily modulate the gut–skin axis by promoting the production of SCFAs (e.g., butyrate, acetate), which regulate immune responses (e.g., IL-10, Treg cells) and enhance skin barrier function. These systemic effects are particularly effective in alleviating conditions like atopic dermatitis and improving hydration. Furthermore, topically applied oligosaccharides, including COSs and GlcOSs, directly interact with skin microbiota and keratinocytes, targeting pathways like MAPK and NF-κB to reduce oxidative stress, inflammation, and UV-induced damage, making them ideal for anti-aging and photoprotection. The distinction underscores how route-specific delivery optimizes therapeutic outcomes—oral for systemic immune modulation and topical for localized skin repair and microbiome balance.

## 5. Commercial Cosmetic Compositions Containing Prebiotic Oligosaccharides

An early patent described the addition of gluco-oligosaccharides (5% by weight, produced by the France company BIOEUROPE) in a series of cosmetic products, including liquid soap, shampoo, body milk (emulsion), face cream (emulsion), and vaginal gel [97]. The gluco-oligosaccharides were prepared from maltose and glucosyltransferase derived from the strain *Leuconostoc mesenteroides* NRLL B-1299, which produced α-(1→6)- and α-(1→2)-linked glucose oligomers with DP in the range of 3–7.

Ecoskin^®^ (Solabia, Pantin, France) is a prebiotic and probiotic complex (in liquid form) formulated with two prebiotic oligosaccharides and probiotic *Lactobacillus* strains (*L. casei* and *L. acidophilus*, with a reported effective dose of 1–5% (*w*/*w*) [128]. The two prebiotic oligosaccharides were α-gluco-oligosaccharides (GlcOSs), produced by enzymatic synthesis from plant substrates (α-glucan oligosaccharides), and β-fructo-oligosaccharides, prepared from Yacon tubers (*Polymnia sonchifolia*) via cold pressing [6]. This patented ingredient was reported to promote the growth and development of beneficial skin commensals and defend the skin from environmental pollutants (up to 2.5 μm) that accelerate aging [129].

Bioecolia^®^ (Solabia, Pantin, France) is a prebiotic product (in powder form) containing α-glucan-oligosaccharides that are produced via enzymatic transglycosylation from saccharose (sucrose) and maltose [130]. Structurally, the α-glucan oligosaccharides are composed of glucose units linked by α-(1→6) and α-(1→2) glycosidic bonds. The specific linkage types allow bioselective, fast, and efficient metabolism of the prebiotic by skin microbes. Specifically, 0.1% Bioecolia^®^ promoted the growth of commensal *S. epidermidis* MFP04 and marginally increased the cytotoxicity of *S. epidermidis* on HaCaT keratinocytes at 1.0% (not enhancing biofilm formation activity) [131]. Bioecolia^®^ was patented for its use as a medication for the treatment of uncomfortable skin/external mucosa and vulvar dryness and/or pruritus and/or vulvar burns [132].

Glycolift^®^ (Solabia, Pantin, France) is a tensing and firming agent containing alginate, α-glucan oligosaccharides, and plant propanediol. Glycolift^®^ is used as an anti-aging tensor by optimizing rheological properties and forming a matrix for instant and prolonged tensing and smoothing action [133]. In addition, it also balances the skin microbiome and acts against atmospheric, UV, and domestic pollutants by forming a non-occlusive matrix. Glycolift^®^ is formulated for facial tensing, reducing wrinkles, and radiant care.

Lancôme^®^, a luxury skincare brand owned by L’Oréal (Paris, France), offers facial serums, sheet masks, eye creams, etc. According to the product label, some Lancôme^®^ products (e.g., facial serums, moisturizers) contain prebiotic α-glucan oligosaccharides that help support a healthy skin microbiome and reduce symptoms of sensitive skin [13]. Other oligosaccharides include trehalose as a moisturizer and maltodextrin as a stabilizer, binding agent, film-forming agent, and skin softener.

Aveeno^®^ (Johnson & Johnson, New Brunswick, NJ, USA) is a clinically validated skincare brand that specializes in dry skin relief, offering products ranging from body washes to moisturizing lotion. Its FDA-approved active ingredient, prebiotic colloidal oatmeal, exhibits multiple benefits in cleansing, buffering, moisturizing, protecting, soothing, anti-irritant, antioxidant, and anti-inflammation properties [134]. Chemically, colloidal oatmeal consists of 65–85% starch, 15–20% protein, 3–11% lipid, 5% fiber, and 5% β-glucan [13]. The high concentrations of starch and β-glucans offer protective and water-holding functions through humectant and film-forming effects, and the presence of different types of phenols confers antioxidant, anti-inflammatory, and UV-absorbing activities [134]. The anti-inflammatory properties, improvement in dry, irritated skin, and enhancement in skin barrier functions have been substantiated and discussed [84,114].

## 6. Conclusions and Future Prospects

This review comprehensively summarized multiple roles of prebiotic oligosaccharides in promoting skin health and their burgeoning applications in the cosmetic industry. Prebiotic oligosaccharides, including human milk oligosaccharides (HMOs), galacto-oligosaccharides (GOSs), fructo-oligosaccharides (FOSs), gluco-oligosaccharides (GlcOSs), and others, have demonstrated significant potential in modulating the skin microbiota, thereby conferring a range of skin health benefits. These benefits encompass the promotion of beneficial skin commensals, the inhibition of pathogenic bacteria, anti-melanogenesis, antioxidant and anti-inflammatory activities, wound healing, the prevention of atopic dermatitis and allergies, enhanced skin hydration, and anti-drying, anti-aging, and photoprotection properties. The mechanisms underlying these benefits are primarily attributed to the selective utilization of prebiotic oligosaccharides by beneficial skin microorganisms, leading to the production of metabolites, such as SCFAs, that modulate the skin environment and immune responses. Additionally, these oligosaccharides can directly inhibit the growth of pathogenic bacteria and exert antioxidant and anti-inflammatory effects through various molecular pathways. The emerging understanding of the structure–function relationships of prebiotic oligosaccharides further highlights their potential for targeted skin health interventions.

Despite the promising findings, several challenges and future directions should be addressed before the practical application of prebiotic oligosaccharides as cosmetic formulations. First, the structure–function relationship of oligosaccharides requires deeper investigation to elucidate how molecular characteristics (monomer composition, DP, glycosidic linkages) dictate their biological activity. This would enable the rational design of novel oligosaccharides from green and sustainable feedstocks (e.g., cellulose, lignocellulosic biomass). Second, rigorous clinical trials are needed to validate the efficacy and safety of prebiotic oligosaccharides. More importantly, mechanistic studies should be devoted to fully understanding the skin–gut microbiota–host interactions and connection to skin health effects. Finally, it is essential to consider the regulatory requirements for cosmetic products and distinguish them from health products. For example, in Europe, cosmetic claim requirements should follow EU Regulation 1223/2009 and 655/2013, while health products should follow European Food Safety Authority (EFSA) guidelines. Standardized protocols should be established for the production and quality control of prebiotic oligosaccharides for application in the cosmetic industry.

## Figures and Tables

**Table 1:** Structural characteristics and production methods of well-established and tentative prebiotic oligosaccharides.

Trivial Names		Abbr.	Monomer Unit(s)	Glycosidic Linkage(s)	DP	Production Methods	Ref.
human milk oligosaccharides		HMOs	Neu5Ac, fucose, galactose, GlcNAc, acetylglucosamine, glucose	β-(1→4), β-(1→6), β-(1→3), α-(1→2), α-(1→3), α-(1→6)	2–10	Glycosyl transfer reaction of sugar nucleotides (e.g., UDP-GlcNAc, GDP-Fuc, and CMP-Neu5Ac) and lactose by glycosyl transferase expressed by modified microorganisms	[31,32,33]
galacto-oligosaccharides		GOSs	galactose, glucose	β-(1→4), β-(1→6), β-(1→3), β-(1→2), β-(1→1) terminal β-(1→4)	2–10	Enzymatic transgalactosylation of lactose by β-galactosidase	[34,35,36]
fructo-oligosaccharides		FOSs	fructose, glucose	β-(2→1), terminal α-(1→2)	2–20 or 2–5	Enzymatic depolymerization of inulin by inulinase; enzymatic transfructosylation from sucrose by β-fructosidase	[37,38,39]
gluco-oligosaccharides	α-glucan oligosaccharides	α-GlcOSs	glucose	α-(1→6), α-(1→4), α-(1→2), α-(1→3), α-(1→1);	2–20	Enzymatic hydrolysis of starch followed by transglycosylation by α-transglucosidase; enzymatic transglycosylation from sucrose by dextransucrase	[40,41]
	β-gluco-oligosaccharides	β-GlcOSs	glucose	β-(1→4)	2–20	Enzymatic/chemical hydrolysis of cellulose or lignocellulosic biomass	[42,43,44,45]
xylo-oligosaccharides		XOSs	xylose	β-(1→4)	2–10	Enzymatic/chemical depolymerization of hemicellulose xylan by β-xylanases	[46]
Chitosan oligosaccharides		COSs	glucosamine	β-(1→4)	2–20	Enzymatic/chemical depolymerization of chitin followed by deacetylation	[47,48]
agaro-oligosaccharides/neoagaro-oligosaccharides		AOSs/NAOSs	alternating 3,6-anhydrogalactose and galactose	α-(1→3), β-(1→4)	2–10	Enzymatic/chemical hydrolysis of agarose (extracted from red algae)	[49,50]

**Table 2:** Summary of in vitro studies of prebiotic oligosaccharides on skin or gut microbiota and associated skin health benefits.

Health Benefits	Oligosaccharides	Effect on Skin Microbiota	Ref.
Promotion of beneficial skin commensals and inhibition of skin pathogens	2′-fucosyllactose (2′-FL, in HMO); GOSs	GOSs (10%, 5%) and 2.5% 2′-FL significantly decreased *Staphylococcus aureus* (SA) and *Pseudomonas aeruginosa* (PA) bacterial growth/CFUs (*p* ≤ 0.05); 1.5% 2′-FL significantly suppressed the lag time of SA growth (*p* ≤ 0.05) and was effective against SA and PA at 2.5% (*p* ≤ 0.01).	[80]
	GOSs	A study found that 5% (*w*/*v*) GOS stimulated *S. epidermidis* DSM 20044 and inhibited *S. aureus* ATCC 25923 both in nutrient broth and cosmetic formulations; higher release rates of GOS oil-in-water (O/W) gel emulsions, with controlled release of GOSs (DP 3) in the hydrogel.	[81]
	FOSs	FOSs (DP 3–5) promoted the growth of beneficial *S. epidermidis* ATCC 12228 while inhibiting both pathogenic *C. acnes* CCUG 1794T and *S. aureus* ATCC 6538 in an in vitro human epithelium model.	[82]
	GlcOSs	A study found that 5% GlcOS inhibited the glycocalyx production by S. aureus cells and suppressed *S. aureus* colonization on the horny cells of atopic dermatitis lesions	[83]
	β-glucan (in colloidal oat)	A study found that 1% colloidal oat (containing 5% β-glucan) promoted the growth rate and metabolism of *S. epidermidis* ATCC 12228 and increased the production of lactic acid (a natural moisturizing factor).	[84]
	Oligochitosan	A study found that 10 kDa oligochitosan showed the highest antimicrobial effect on *C. acnes* with a minimum inhibitory concentration of 32–64 μg/mL; it also worked synergistically with antibiotics (tetracycline or erythromycin) against *C. acnes*.	[85]
	FOSs, GOSs, IMOs, inulin	FOSs, GOSs, IMOs, and inulin significantly promoted the in vitro growth of *S. epidermidis* CCSM0287 and increased SCFA production; the addition of 2% FOSs post-fermentation supernatant significantly inhibited *S. aureus* CCSM0424 biofilm formation.	[23]
	FOSs, GOSs, IMOs, LAG	A study found that 1% GOS/FOS/IMO/LAG with 1% xylitol showed species-specific antibacterial/antibiofilm against pathogenic *S. aureus* strains while not affecting *S. epidermidis* strains.	[86]
	Oligosaccharides derived from *Ulva* sp. (green alga)	Oligosaccharides derived from *Ulva* sp. (1.5 kDa, 8 kDa) at 1000 μg/mL did not significantly modify the cytotoxicity of *S. aureus* MFP03 and *S. epidermidis* MFP04 towards HaCaT keratinocytes in vitro, but they could modify the bacterial biofilm structures; the oligosaccharides induced a decreased inflammatory potential of both acne and non-acne *C. acnes* strains on keratinocytes of up to 39.8%.	[87]
Anti-melanogenesis	2′-fucosyllactose	An MTT assay performed on MNT-1 cells and human-derived melanocytes showed a 40% reduction in melanin production from 2′-FL treatment (no cytotoxicity at 20 g/L or less), which was mediated through autophagy activation and inhibited the expression of melanogenesis proteins TYR and TYRP1.	[88,89]
	GOSs	GOSs (74.9% purity) at 70 mg/mL or lower concentrations showed no cytotoxicity in B16F10 melanoma cells in vitro; melanin accumulation was inhibited at 14 mg/mL GOS or higher; 14–35 mg/mL GOS showed a protective effect on HaCaT keratinocytes upon UVB irradiation.	[29]
	AOSs, NAOSs	3,6-anyhydro-L-galactose (AHG, prepared from enzymatic hydrolysis of agarose) at a concentration of 100 μg/mL showed significantly lower melanin production compared to arbutin in an in vitro skin whitening assay; 100/200 μg/mL AHG showed strong anti-inflammatory activity in suppressing nitrite production.	[90]
	AOSs, NAOSs	AOSs, NAOSs, and AHG at 50 μg/mL did not show significant cytotoxicity in murine B16 melanoma cells (B16F10) and human epidermal melanocytes (HEMs) in vitro; AHG and NAOSs (DP 4, DP 6) at 50 μg/mL both significantly inhibited the α-melanocyte-stimulating hormone (α-MSH)-induced melanin production in B16F10 cells and HEMs (AHG highest), while AOSs of DP 3, DP 5, and DP 7 (50 μg/mL) did not; AHG is the monomer unit responsible for the anti-melanogenic effect of agar-derived oligosaccharides.	[91]
Antioxidant and anti-inflammation	AOSs, NAOSs	A mixture of AHG and AOSs (DP 3) showed significantly higher reactive oxygen species (ROS)-scavenging activity than NAOSs (DP 2 and DP 4) in H_2_O_2_-induced human dermal fibroblasts (HDFs) in vitro, exerting ability in protecting skin from oxidative stress (antioxidant activity); AHG and AOSs (DP 3) exhibited no cytotoxicity in HDFs and promoted cell proliferation.	[92]
	α-gluco-oligosaccharides (α-GlcOSs)	α-GlcOSs (average Mw 1137 Da) with 24% α-(1→4) and 76% α-(1→6) linkages exhibited a prebiotic effect on six bacterial and yeast strains and anti-inflammatory activity towards RAW 265.7 macrophage cells by suppressing the expression of nitric oxide synthase, tumor necrosis factor-α, IL-1β, IL-6, and IL-10 and inhibiting the nuclear factor-kappa B (NF-κB) signaling pathways.	[93]
	pectic oligosaccharides (POSs)	POSs derived from the Fenton hydrolysis of okra pectin showed improved antioxidant and anti-inflammatory activities compared with okra pectin; POSs of 1.79 kDa (lowest Mw) showed the highest antioxidant activity by scavenging DPPH and ABTS radicals and also the highest anti-inflammatory activity by inhibiting LPS-induced NO production, iNOS expression, and expression of IL-1β and IL-6 mRNAs by suppressing NF-κB signaling pathways in RAW 264.7 cells.	[94]
Wound healing	GOSs	GOSs (prepared from whey permeate via fermentation by *Lactobacillus delbrueckii* ssp.) promoted wound healing and skin health by its direct activity on keratinocyte functions, including stimulation on the beneficial reversible inflammatory response, mitochondrial respiration, cell migration, and differentiation in HaCaT cells in vitro.	[30]
	GOSs, inulin	A diet supplemented with GOSs or inulin improved the general health of mice undergoing surgical colonic anastomosis, indicated by increased body weight and induced thickening of the colonic wall; increased butyrate production and better anastomotic healing was observed, with enhanced mucosal continuity.	[95]
	Thiolated chitosan oligosaccharides/hyaluronic acid oligosaccharide-based hydrogels	Thiolated poly- and oligosaccharide-based hydrogels were found to have good biocompatibility, biodegradability, and nontoxicity, and favorable for wound healing processes, such as in situ gelling, cell adhesion (bioadhesion), drug release controlling, enzyme inhibitory, and metal binding properties (mitigate toxic effects).	[96]

Notes: GOSs: galacto-oligosaccharides; FOSs: fructo-oligosaccharides; GlcOSs: gluco-oligosaccharides; IMOs: isomalto-oligosaccharides; LAG: arabinogalactan; AOSs: agaro-oligosaccharides; NAOSs: neoagaro-oligosaccharides; AHG: 3,6-anyhydro-_L_-galactose; DP: degree of polymerization.

**Table 3:** Summary of in vivo studies and clinical trials of prebiotic oligosaccharides on the skin–gut microbiota and skin health.

Functions	Treatment	Effect on Skin–Gut Microbiota and Skin Health	Trial Type	Ref.
Alleviation/prevention of atopic dermatitis and allergies	Oral HMOs	HMO metabolism in infants helps establish the infant gut microbiota, modulate the immune system, and prevent against atopic dermatitis (AD)/eczema development or reduce the severity through the regulation of the immune response (e.g., IL-10)	human trial	[17,105]
	Oral GOSs	Dietary GOS treatment (0.5 or 1.5 g/kg) on DNFB-induced AD mice (N = 40) markedly relieved skin inflammation by decreasing IgE, IL-4, IL-13, IFN-γ, and TNF-α production and regulating PPAR-γ/NF-κB signaling; GOS treatment also significantly improved anxiety- and depressive-like symptoms by normalizing the neurotransmitter levels in the brain	mice studies	[18]
	Oral FOSs	Oral intake of kestose-rich FOSs (>85% kestose, 7.3 mg/day) alleviated atopic dermatitis in ovalbumin-sensitized Balb/c mice (N = 42) by modulating the gut–skin axis (altered the intestinal microbiome and significantly increased butyric and propionic acid levels in mice feces) and immune response (markedly decreased IgE levels and regulatory T cell-mediated T helper cell type 2, mast cells, and eosinophils)	mice studies	[106]
	Oral alginate oligosaccharides (AOSs)	Oral gavage (10 mg/kg body weight) of AOSs in natural aging mice caused modulation of the colonic butyrate-HIF-1α axis homeostasis, which promoted entry of butyrate into the skin and upregulated the mitophagy level, thus improving skin aging via the HDAC3/PHDHIF-1α/mitophagy loop in the skin of mice	mice studies	[107]
Attenuation of acne vulgaris	Oral FOSs and GOSs	Consecutive intake (3 months) of FOSs (100 mg) and GOSs (500 mg) in female adults with acne improved glycemic and lipid metabolism	human trials (N = 12)	[108]
	Oligochitosan (chitosan oligosaccharides)	A study found that 10 kDa oligochitosan showed the highest antimicrobial effect, with minimum inhibitory concentration values of 32–64 μg/mL on *C. acnes*; combination of tetracycline– or erythromycine–10 kDa oligochitosan resulted in a median Σ fractional inhibitory concentration (FIC) range of 0.02–0.56	in vitro and in vitro	[85,109]
	Seaweed oligosaccharides with zinc complex (SOZC)	A study found that 5.6% SOZC reduced *C. acnes* counts by 74% in vitro, and SOZC was found to have soothing effects, indicated by increased levels of interleukin 1 alpha by 11.1%; a gel containing 5% SOZC significantly improved acne vulgaris symptoms by 14 days with reduced sensitivity and improved skin healing	double-blind human trials (N = 12)	[110]
Skin hydration, anti-dryness	Topical GOSs	GOS treatment (in a cosmetic serum) on healthy females (40–60 yrs) significantly improved skin water-holding capacity (positively correlated with *Enhydrobacter*), reduced transepidermal water loss (TEWL, negatively correlated with *Enterobacteriaceae*), erythema index, wrinkle depth, and *S. aureus* populations	double-blind human trials (N = 60)	[24,25]
	Oral GOSs	Oral administration of GOSs (100 mg, 12 weeks) on hairless mice increased the water-holding capacity of the skin, prevented transepidermal water loss, and reduced erythema formation (16.8%)	mice studies	[111]
	Oral GOSs with *B. breve*	Daily intake of fermented milk containing prebiotic GOSs and probiotic *B. breve* (YIT 12272, Yakult) for 4 weeks was found to benefit skin condition through a reduction in serum total phenol levels; it also prevented skin dryness (increased hydration level of stratum corneum) and disrupted keratinization in healthy adult women (N = 40)	a double-blind, placebo-controlled, randomized human trial	[28,112]
	Topical α-gluco-oligosaccharides (α-GlcOSs)	Topical application of α-GlcOSs (in a lotion, 4 weeks) and collagen tripeptide F in females with sensitive atopic skin (N = 40, 30–59 yrs) significantly reduced TEWL and improved skin hydration, roughness, desquamation, and skin irritability	a double-blind, placebo-controlled, randomized human trial	[113]
Anti-itching	Oral FOSs	Oral take of FOSs (4.25 g 1-kestose) for 12 weeks alleviated itching and sleep disturbance symptoms in children (N = 48, 2–17 yrs) with atopic dermatitis while inducing specific alterations in the skin microbiome (decreased *Lachnospiraceae* abundance; increased *Prevotella* and *Rothia* abundance) and epidermal lipid profiles	a randomized, double-blind, placebo-controlled human trial	[19]
	Topical colloidal oat (starch and β-glucans)	Topical application (6-week, twice a day) of a moisture containing 1% colloidal oat on female subjects with dry skin significantly increased the concentration of lactic acid (a natural moisturizing factor for stratum corneum); 1% colloidal oat altered the gene expression profile of *S. epidermidis*, alleviating skin dryness	in vitro and human trials	[84]
	Topical colloidal oatmeal (containing 5% β-glucans, 5% fiber)	Two-week application (twice a day) of a colloidal oatmeal skin protectant lotion on healthy females (N = 29) with itchy dry skin on their lower legs showed significant improvement in skin dryness, scaling, roughness, and itch intensity, potentially attributed to the antioxidant and anti-inflammatory activities of colloidal oatmeal	a blind human trial	[114]
Anti-aging and photoprotection	Oral GOSs	Oral intake of GOSs (200 mg/kg) increased TEWL, decreased water-holding capacity, and significantly reduced the wrinkle area and mean wrinkle length in hairless mice exposed to UVB; inflammatory cytokines (IL-6, IL-12, TNF-α) induced by UVB were significantly reduced; UVB-induced MAPK phosphorylation of kinases (JNK, p38, ERK) contributing to skin aging was significantly inhibited	mice studies	[115]
	Oral GOSs with *B. longum*	Oral treatment of GOSs with *B. longum* protected the skin of hairless mice against UVB-induced photo-aging and showed anti-inflammatory and antioxidative effects	mice studies	[111,116]
	Oral GOSs with collagen tripeptide (CTP)	Oral administration of CTP and GOS mixtures (different ratios) in UVB-irradiated hairless mice (8 weeks) showed an inhibitory effect on photo-aging, including a reduction in photoaged physical parameters (TEWL, wrinkle area) and serum levels of pro-inflammatory cytokines; the treatment also reversed the increasing abundance of Firmicutes induced by UVB and increased the *Verrucomicrobiota* abundance	mice studies	[117]
	Topical α-glucan oligosaccharides	Treatment (28 days) with a moisturizer formulated with α-glucan oligosaccharides and postbiotics (*Pseudoalteromonas* ferment extract) positively influenced skin microbiome diversity and balance and provided anti-aging benefits to females (N = 25, 35–65 yrs) with Fitzpatrick skin types I–VI, moderate crow’s feet wrinkles, and global face photodamage	a human trial followed EU Scientific Committee on Consumer Safety (SCCS) protocol	[26]
	Oral and/or topical xylo-oligosaccharides (XOSs)	Oral and/or topical application of XOSs regulated facial cutaneous aging in females (N = 77) by inhibiting *Cutibacterium* abundance and the enrichment of intestinal *Bifidobacterium*	a double-blind placebo-controlled human trial	[21]
	Topical chitosan oligosaccharides (COSs)	Topical application (10 weeks) of COSs (average Mw ≤ 1000 Da, 50/100/200 mg/mL) on hairless mice (N = 12) contributed to the prevention of UV-induced skin dryness, epidermal hyperplasia, wrinkle formation by increasing activities of antioxidative enzymes, and suppressing the production of pro-inflammatory cytokines	mice studies	[27]
	Topical succinyl-chitosan oligosaccharides (SU-COSs)	SU-COSs (6400 Da, substitution degree 69.26%) exhibited good biocompatibility and promoted the migration of HaCaT cells in vitro; in vitro treatment of SU-COSs exerted a protective effect on UVB-damaged HaCaT cells by preserving cytoskeletal morphology, regulating cell cycle, scavenging intracellular reactive oxygen species, and inhibiting apoptosis; in vivo treatment of SU-COSs repaired UVB-damaged mice skin by reducing skin erythema and water loss, relieving crusting, and maintaining skin epidermis thickness the stability of collagen fibers	in vitro and mice studies	[22]
	Topical ginseng oligosaccharide extract (GSOs)	GSO (1000 Da, β-linkages) treatment (0.2/1.0/2.0 mg/cm^2^/day) on the dorsal skin of BALB/c hairless mice (N = 25) attenuated UVB-induced epidermal thickening and moisture loss; GSOs improved the expression of skin barrier proteins (FLG, IVL, AQP3) related to skin dryness and the enzymes involved in stratum corneum exfoliation (increased DSG1, decreased KLK7)	mice studies	[118]

**Table 4:** Mechanism of action of different oligosaccharides exerting skin health benefits.

Pathway	Oligosaccharides	Administration Route	Mechanism of Action	Skin Health Benefits
AMPK–ULK1	HMOs (2′-FL)	oral	Activates AMPK, phosphorylates ULK1, induces autophagy, and downregulates melanogenic enzymes (TYR, TYRP1).	Anti-melanogenesis
MAPK	GOSs	oral	Inhibits UVB-induced MAPK (JNK/p38/ERK), reducing wrinkle formation and inflammation.	Anti-aging, photoprotection, anti-wrinkle
	COSs	topical	Suppresses MAPK signaling, scavenges ROS, and protects against UV damage.	Photoprotection, anti-aging
	XOSs	oral, topical	Modulates *Cutibacterium* abundance and lipid synthesis via MAPK.	Anti-aging, microbiome balance
NF-κB	GOSs	oral	Downregulates NF-κB, reducing pro-inflammatory cytokines (IL-6, TNF-α).	Anti-atopic dermatitis, anti-inflammation
	FOSs	oral	Enhances SCFA production, indirectly suppressing NF-κB in the gut–skin axis.	Skin hydration, itch relief
	AOSs (alginate)	oral	Upregulates the butyrate–HIF-1α axis, inhibiting NF-κB-driven inflammation.	Anti-aging, skin repair
	GlcOSs	topical	Blocks NF-κB activation in keratinocytes, reducing oxidative stress.	Anti-inflammatory, sensitive skin relief
SCFAs	HMOs	oral	Gut microbiota metabolize HMOs to SCFAs (butyrate), promoting Treg cells and IL-10.	Prevents infant atopic dermatitis
	GOSs	oral	Increases fecal SCFAs (acetate/butyrate), modulating the gut–skin immune response.	Anti-eczema, anxiolytic effects
	FOSs	oral	Boosts *Lactobacillus*-derived SCFAs, improving skin barrier lipids.	Reduces transepidermal water loss (TEWL)

## Data Availability

All data and materials are included in this manuscript.
